# Neuronal GRK2 regulates microglial activation and contributes to electroacupuncture analgesia on inflammatory pain in mice

**DOI:** 10.1186/s40659-022-00374-6

**Published:** 2022-02-03

**Authors:** Yu Chen, Yang Zhou, Xiao-Chen Li, Xue Ma, Wen-Li Mi, Yu-Xia Chu, Yan-Qing Wang, Qi-Liang Mao-Ying

**Affiliations:** 1 Department of Integrative Medicine and Neurobiology, School of Basic Medical Sciences, Shanghai Medical College, Institute of Acupuncture Research, Institutes of Integrative Medicine, Fudan University, Shanghai, 200032 People’s Republic of China; 2 State Key Laboratory of Medical Neurobiology and MOE Frontiers Center for Brain Science, Institutes of Brain Science, Fudan University, Shanghai, 200032 People’s Republic of China; 3 Shanghai Key Laboratory of Acupuncture Mechanism and Acupoint Function, Fudan University, Shanghai, 200433 People’s Republic of China

**Keywords:** Inflammatory pain, Microglial activation, Neuroinflammation, Electroacupuncture analgesia, G protein coupled receptor kinase 2

## Abstract

**Background:**

G protein coupled receptor kinase 2 (GRK2) has been demonstrated to play a crucial role in the development of chronic pain. Acupuncture is an alternative therapy widely used for pain management. In this study, we investigated the role of spinal neuronal GRK2 in electroacupuncture (EA) analgesia.

**Methods:**

The mice model of inflammatory pain was built by subcutaneous injection of Complete Freund’s Adjuvant (CFA) into the plantar surface of the hind paws. The mechanical allodynia of mice was examined by von Frey test. The mice were subjected to EA treatment (BL60 and ST36 acupuncture points) for 1 week. Overexpression and downregulation of spinal neuronal GRK2 were achieved by intraspinal injection of adeno associated virus (AAV) containing neuron-specific promoters, and microglial activation and neuroinflammation were evaluated by real-time PCR.

**Results:**

Intraplantar injection with CFA in mice induced the decrease of GRK2 and microglial activation along with neuroinflammation in spinal cord. EA treatment increased the spinal GRK2, reduced neuroinflammation, and significantly decreased CFA-induced mechanical allodynia. The effects of EA were markedly weakened by non-cell-specific downregulation of spinal GRK2. Further, intraspinal injection of AAV containing neuron-specific promoters specifically downregulated neuronal GRK2, and weakened the regulatory effect of EA on CFA-induced mechanical allodynia and microglial activation. Meanwhile, overexpression of spinal neuronal GRK2 decreased mechanical allodynia. All these indicated that the neuronal GRK2 mediated microglial activation and neuroinflammation, and subsequently contributed to CFA-induced inflammatory pain.

**Conclusion:**

The restoration of the spinal GRK2 and subsequent suppression of microglial activation and neuroinflammation might be an important mechanism for EA analgesia. Our findings further suggested that the spinal GRK2, especially neuronal GRK2, might be the potential target for EA analgesia and pain management, and we provided a new experimental basis for the EA treatment of pain.

**Supplementary Information:**

The online version contains supplementary material available at 10.1186/s40659-022-00374-6.

## Background

It has been well known that acupuncture analgesia is essentially the result of the interaction of nociceptive signals from the pain regions and acupuncture triggered signals from the acupoints (the specific somatic tissues also called acupuncture points) at different levels along with nociceptive pathway in central nervous system (CNS) [1, 2]. A large number of classical transmitters, endogenous opioids, non-classical modulators as well as non-neuronal cells are thought to be involved in the process of acupuncture analgesia [1, 3]. Though great progress has been made on the research of acupuncture analgesia in the past decades, knowledge on the mechanisms underlying electroacupunture (EA) analgesia still needs to be further deepened.

G protein coupled receptor kinase 2 (GRK2) is a serine/threonine kinase that restrains signaling by promoting the G protein coupled receptors (GPCRs) desensitization or internalization [4] and/or by interacting with multiple components of intracellular signaling pathways [5–7]. It can protect cells against overstimulation [8, 9]. It has been reported that GRK2 protein level can be substantially reduced in peripheral blood mononuclear cells of rheumatoid arthritis or multiple sclerosis patients [10, 11]. Evidence from the animal investigation showed that inflammatory pain in rodent models was associated with the decreased level of GRK2 expression in the spinal cord [12–16]. Our previous study has demonstrated that downregulation of spinal GRK2 by intrathecal exposure of GRK2 antisense completely eliminated both transient and persistent antiallodynic effect by EA treatment on inflammatory pain [17]. This indicated that the spinal GRK2 played an important role in EA analgesia on inflammatory pain. However, the role of spinal neuronal GRK2 in EA analgesia on inflammatory pain is still unclear.

The microglia were thought to be a kind of immune cells located in the central nervous system [18–21]. Studies have found that microglia in the spinal cord were significantly activated in various pain models [22–27]. The inhibition of microglial activation can relieve inflammatory pain, neuropathic pain, cancer pain and chemotherapy-induced peripheral neuropathy [25–28]. It is well known that pro-inflammatory microglia (CD16/32 as marker) and pro-inflammatory cytokines, including interleukin (IL) -1β and tumor necrosis factor (TNF)-α are involved in the process of neuroinflammation [29, 30]. While anti-inflammatory microglia (CD206 as marker) and anti-inflammatory cytokines, including transforming growth factor (TGF) -β, IL-10 and IL-4 also play a role in inflammatory response [31].

The previous study has proved that electroacupuncture can inhibit the level of OX-42 (a marker of microglia cells) as well as the release of pro-inflammatory cytokines IL-1, IL-6 and TNF-α in Complete Freund’s adjuvant (CFA)-induced inflammatory pain in rats [32], suggesting that EA might have a regulatory effect on microglial activation and neuroinflammation in the spinal cord. However, in inflammatory pain, whether the regulatory effect of EA on spinal microglial activation and neuroinflammation is dependent on neuronal GRK2 in the spinal dorsal horn or not still remains unknown.

Therefore, we hypothesized that in mice model of inflammatory pain, restoration of the spinal GRK2 and subsequent microglial activation and neuroinflammation in the spinal cord are important mechanisms for EA analgesia, and that the neuronal GRK2 in the spinal cord might be an important target contributed to EA analgesia. To test our hypothesis, we first observed the expression of GRK2 and microglial activation in the spinal cord in CFA- and EA-treated mice. To evaluate the importance of GRK2 in CFA-induced inflammatory pain and EA analgesia, an AAV vector delivering GRK2 shRNA was used to downregulate GRK2 in the spinal cord. Finally, in order to investigate the possible neuronal GRK2 and microglial interaction, an AAV vector with neuronal promotor was designed to up-regulate or downregulate the neuronal GRK2 in this study. To our knowledge, this is the first study of spinal cord GRK2 on mice model of inflammatory pain, highlighting the importance of spinal GRK2, especially the neuronal GRK2 in the spinal cord in EA analgesia.

## Results

### Repeated EA reduced inflammatory pain, restored GRK2 expression and suppressed microglial activation in the spinal cord

**Fig. 1:**
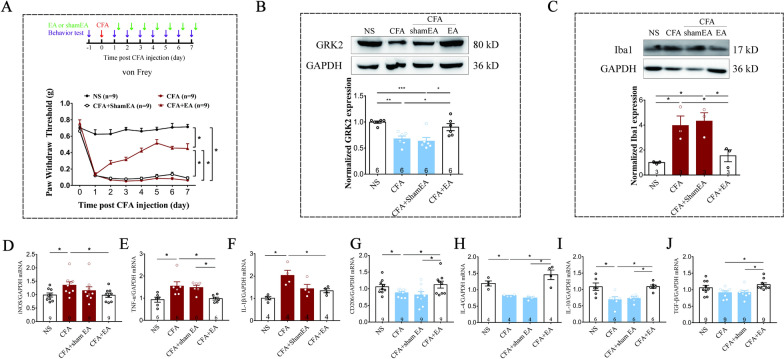
Repeated EA reduced inflammatory pain and regulated microglial activation. **A** Repeated EA treatment statistically increased the decreased mechanical threshold induced by CFA. Values are represented as mean ± SEM. n = 9 for normal saline group, CFA group, CFA + EA group and CFA + shamEA group. The statistical method is repeated measures ANOVA. *p < 0.05. **B** Western blot analysis of GRK2 after EA. EA treatment reversed the decline of spinal GRK2 induced by CFA. Results are normalized to GAPDH and shown as ratios to saline-treated mice. Values are represented as mean ± SEM. *p < 0.05, **p < 0.01, ***p < 0.001. **C** EA treatments suppressed the expression of Iba1 at the 7th day after the CFA injection. Results are normalized to GAPDH shown as ratios to NS (saline-treated) mice. Values are represented as mean ± SEM. *p < 0.05. The statistical method is one-way ANOVA multiple comparisons. **D**–**F** Real-time PCR analysis showed that the mRNA expression of iNOS, TNF-α, IL-1β were up-regulated after the CFA injection and inhibited by EA. **G**–**J** Real-time PCR analysis showed that the mRNA expression of CD206, IL-4, IL-10 were remarkably decreased by CFA and up-regulated by EA. Though TGF-β only showed a trend of decline after the CFA injection. Results are normalized to GAPDH mRNA. Values are represented as mean ± SEM. *p < 0.05. The statistical method is one-way ANOVA multiple comparisons

To observe the effect of EA on CFA-induced inflammatory pain, EA or sham EA was administered once a day after the behavior tests, starting from the second day after the CFA injection (Fig. 1A). Similar to the previous report [33], the mice exhibited a significant decline of mechanical paw withdrawal threshold 24 h after the CFA injection (n = 9 for normal saline (NS) group, CFA group, CFA + EA group and CFA + shamEA group) (*p < 0.05). EA treatment markedly alleviated CFA-induced mechanical allodynia (*p < 0.05), but sham EA had no significant effect on it (*p > 0.05, Fig. 1A).

The protein levels of GRK2 have been demonstrated to be key factors in regulating persistent inflammatory pain [15], and the decreased GRK2 was thought to be associated with the transition from acute pain to chronic pain [13]. Our results showed that the expression of GRK2 decreased in the spinal cord from the 1st to the 7th day after the CFA injection according to our supplemental results (*p < 0.05, **p < 0.01, Additional file 1: Fig. S1). At the 7th day after the CFA injection, EA treatment markedly increased the protein levels of GRK2 in the spinal cord (n = 6 for normal saline group, CFA group, CFA + EA group and CFA + shamEA group) (*p < 0.05, **p < 0.01, Fig. 1B) compared with either CFA− or CFA+ shamEA -treated groups.

The activation of microglia in the spinal cord has been demonstrated to play an irreplaceable role in the neuroinflammation [19]. The western blot analysis showed that EA treatment noticeably reduced the CFA-upregulated Iba1 (a marker of microglia cells) in the spinal cord after the CFA injection (*p < 0.05, Fig. 1C), compared with either CFA− or CFA+ shamEA -treated groups. Further analysis by qPCR uncovered that the mRNA expression of inducible nitric oxide synthase (iNOS) as well as pro-inflammatory cytokines (TNF-α, IL-1β) were up-regulated after the CFA injection (*p < 0.05) and that EA inhibited these up-regulation in the spinal dorsal horn (Fig. 1D–F), compared to CFA-treated groups. On the contrary, CFA remarkably decreased the mRNA expression of CD206 (anti-inflammatory microglial marker [30]) (*p < 0.05) and anti-inflammatory cytokines (IL-4, IL-10) (*p < 0.05), and that EA up-regulated them in this study (*p < 0.05, Fig. 1G–J), compared to CFA + shamEA-treated groups. Though the mRNA expression of TGF-β only showed a trend of decline after the CFA injection (p = 0.11), EA significantly up-regulated the level of TGF-β in EA treated mice as compared to the CFA-treated mice or the sham EA treated mice (*p < 0.05, Fig. 1J). These results indicated that EA played its regulatory effect on microglial activation and inhibited the spinal neuroinflammation in the CFA-induced pain model.

### EA induced analgesia by restoring GRK2 expression and regulating inflammatory response

**Fig. 2:**
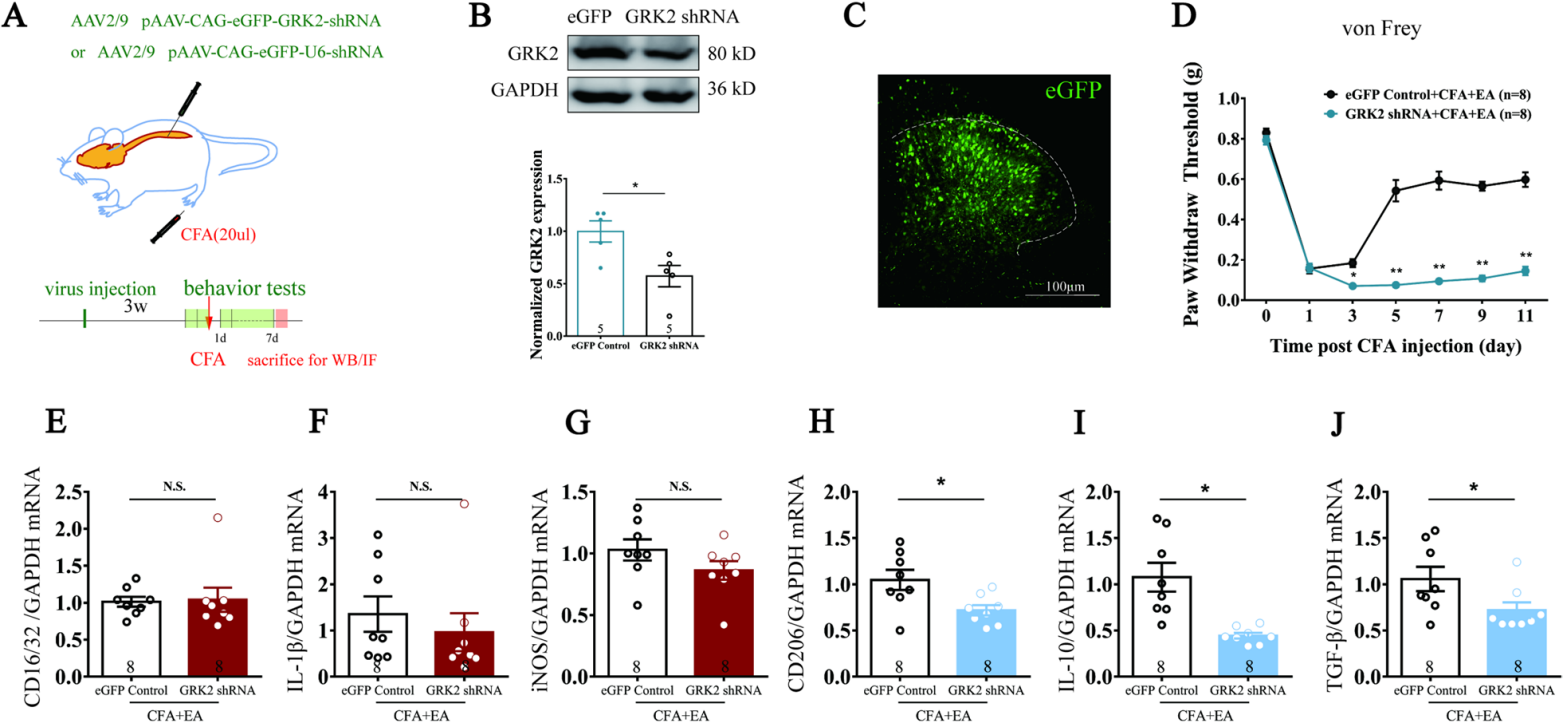
GRK2 involved in EA analgesia and neuroinflammation reduction. **A** Experimental strategy for inhibiting spinal cord GRK2 expression by virus injection. **B** Western blot analysis showed that GRK2 protein expression was downregulated in the spinal dorsal horn of the mice 3 weeks after the virus administration. Results are normalized to GAPDH and shown as ratios to eGFP control group. Values are represented as mean ± SEM. *p < 0.05 versus eGFP control group. Student’s *t* test was used for comparisons. **C** Representative images of the expression of eGFP in the spinal cord at the third week after AAV administration to the spinal cord, scale bar = 100 μm. **D** Downregulation of GRK2 in the spinal cord eliminated the analgesic effect of EA in CFA-induced mechanical allodynia. Values are represented as mean ± SEM. *p < 0.05, **p < 0.01, ***p < 0.001. The statistical method is two-way ANOVA multiple comparisons. **E**–**G** Real-time PCR analysis showed that the mRNA expression of CD16/32, iNOSIL-1β had no significant change. **H**–**J** Real-time PCR analysis showed the increase of the mRNA expression of CD206, IL-10, TGF-β by EA was inhibited by the downregulation of GRK2. Results are normalized to GAPDH mRNA. Values are represented as mean ± SEM. *p < 0.05. Student’s *t* test was used for comparisons

To further confirm the role of spinal GRK2 in EA analgesia, an AAV virus vector delivering GRK2 shRNA drived by CAG (a ubiquitous promoter used to drive high levels of gene expression in mammalian cells), was designed and injected into the spinal dorsal horn in the present study (Fig. 2A). The AAV injection significantly down-regulated the GRK2 protein level in the spinal cord in pAAV-CAG-eGFP-GRK2-shRNA (GRK2 shRNA for short) group, compared to control group pAAV-CAG-eGFP-U6-shRNA (eGFP for short) (n = 5 for eGFP group and GRK2 shRNA group, *p < 0.05, Fig. 2B). The virus was visualized by the green fluorescence and was located in the spinal dorsal horn according to the fluorescence microscope (Fig. 2C). The downregulation of GRK2 in the spinal cord by AAV injection did not alter locomotor activity and sensitivity of mechanical stimulation in naive mice according to our supplemental results (p > 0.05, Additional file 1: Fig. S2), but it significantly prevented EA analgesia on mechanical allodynia (*p < 0.05, **p < 0.01, ***p < 0.001, Fig. 2D) in the CFA-treated mice (n = 8 for eGFP + CFA + EA group and GRK2 shRNA + CFA + EA group). Furthermore, the down-regulation of spinal GRK2 abolished the EA-induced up-regulation of CD206 (*p < 0.05, Fig. 2H) and IL-10, TGF-β mRNA (*p < 0.05, Fig. 2I, J), but it had no significant effect on CD16/32 (p > 0.05, Fig. 2E, G) and iNOS, IL-1β (p > 0.05, Fig. 2F).

### Neuronal GRK2 in the spinal cord contributed to EA analgesia

**Fig. 3:**
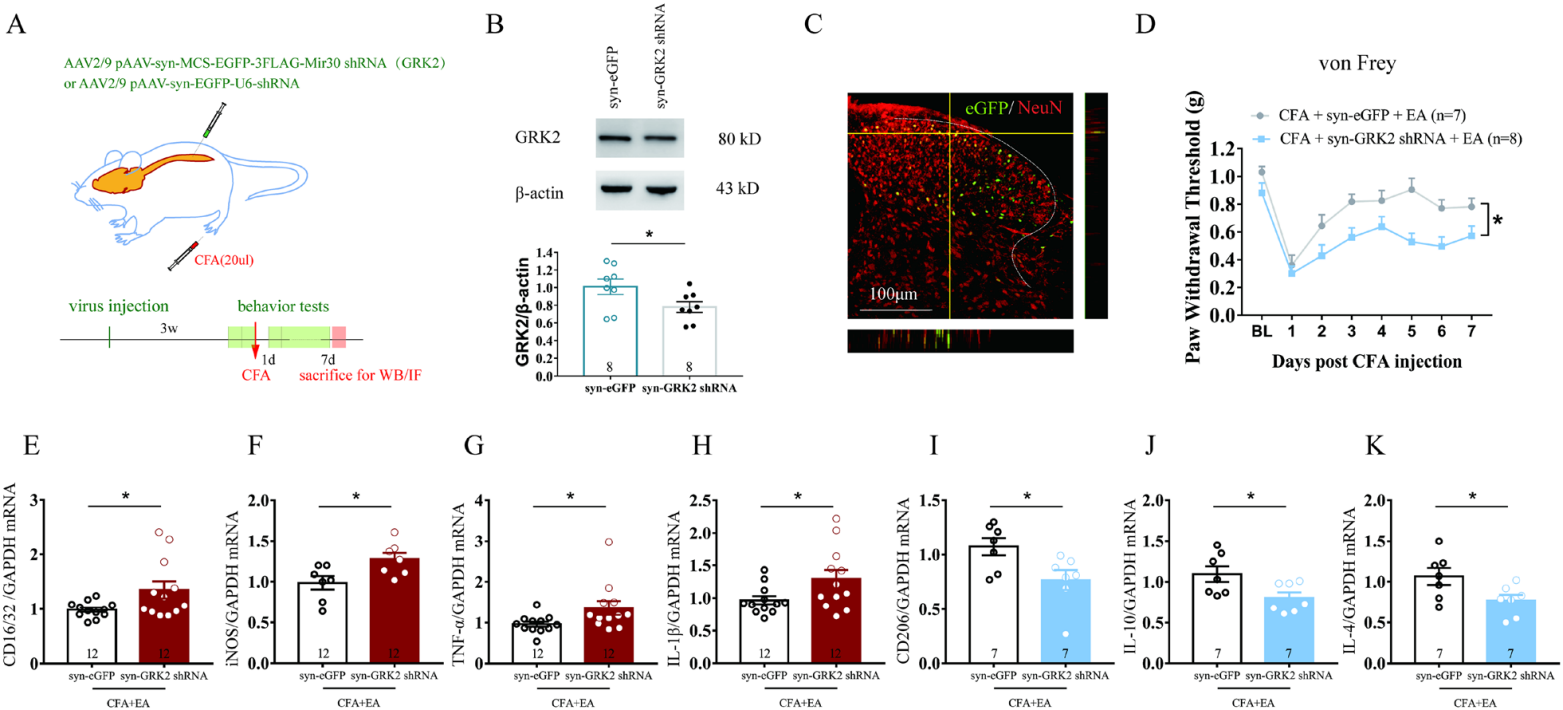
Neuronal GRK2 in the spinal cord contributed to EA analgesia. **A** Experimental strategy for inhibiting spinal cord neuronal GRK2 expression by neuron-specific promoter Syn. **B** Western blot analysis of downregulated GRK2 protein expression in the spinal dorsal horn of mice 3 weeks after the virus administration. n = 8 for syn-eGFP group and syn-GRK2 shRNA group. Student’s *t* test was used for comparisons. *p < 0.05. **C** Representative images showed NeuN labeling (red) and eGFP-tagged (green) AAV-shRNA in the spinal cord. Scale bar, 100 μm. **D** Downregulation of neuronal GRK2 in the spinal cord inhibited the analgesic effect of EA in CFA-induced mechanical allodynia. Data are expressed as means ± SEM. n = 7 for syn-eGFP + CFA + EA group and n = 8 for syn-GRK2 shRNA + CFA + EA group. The statistical method is repeated measures ANOVA. *p < 0.05. **E**–**H** qRT-PCR showed neuron-specific downregulation of GRK2 inhibited the decrease of the mRNA expression of CD16/32, iNOS, TNF-α, IL-1β by EA. *p < 0.05. Student’s *t* test was used for comparisons. **I**–**K** Downregulation of neuronal GRK2 inhibited the rise of the mRNA expression of CD206, IL-10, IL-4by EA treatment. Results are normalized to GAPDH mRNA. Data are expressed as means ± SEM. *p < 0.05. Student’s *t* test was used for comparisons. n = 7 for syn-eGFP + CFA + EA group and syn-GRK2 shRNA + CFA + EA group

Neuronal GRK2 in DRG has been documented to prevent the prolongation of epinephrine-induced hyperalgesia and CFA-induced pain in mice [13, 15]. In order to evaluate whether neuronal GRK2 in the spinal cord contributes to EA analgesia on inflammatory pain and EA regulation on neuroinflammation, the mice with low GRK2 expression in the spinal cord neuron was modeled by intraspinal injection with an AAV vector delivering GRK2 shRNA with a neuronal specific promoter Syn (synapsin) (Fig. 3A), pAAV-syn-MCS-EGFP-3FLAG-Mir30shRNA (Grk2) (syn-GRK2 for short) and control group pAAV-syn-EGFP-U6-shRNA (syn-eGFP for short). The AAV virus injection decreased the GRK2 level in the spinal cord (*p < 0.05, Fig. 3B) (n = 8 for syn-eGFP group and syn-GRK2 shRNA group). The AAV expressed eGFP (green) was collocated with NeuN labeled (red) neurons (Fig. 3C). The injection sites were located in the superficial dorsal horn of the spinal cord, which can be regulated by nociceptive afferents [34–37]. Further, downregulation of GRK2 level in spinal neuron did not affect the locomotor activity and the sensitivity of mechanical stimulation in naive mice according to our supplemental results (p > 0.05, Additional file 1: Fig. S3). In the CFA-treated mice, the downregulation of neuronal GRK2 significantly inhibited EA analgesia on mechanical allodynia (*p < 0.05, Fig. 3D) (n = 7 for syn-eGFP + CFA + EA group and n = 8 for syn-GRK2 shRNA + CFA + EA group). Neuron-specific downregulation of GRK2 significantly weakened the down-regulatory effect of EA on CD16/32, iNOS, TNF-α, IL-1β (*p < 0.05, Fig. 3E–H) and it also weakened the up-regulatory effect of EA on CD206 and IL-10, IL-4 (*p < 0.05, Fig. 3I–K) (n = 7 for syn-eGFP + CFA + EA group and syn-GRK2 shRNA + CFA + EA group). These results suggested that downregulation of neuronal GRK2 in the spinal cord significantly inhibited the regulation of EA on microglial activity and spinal cord neuroinflammation after CFA injection.

**Fig. 4:**
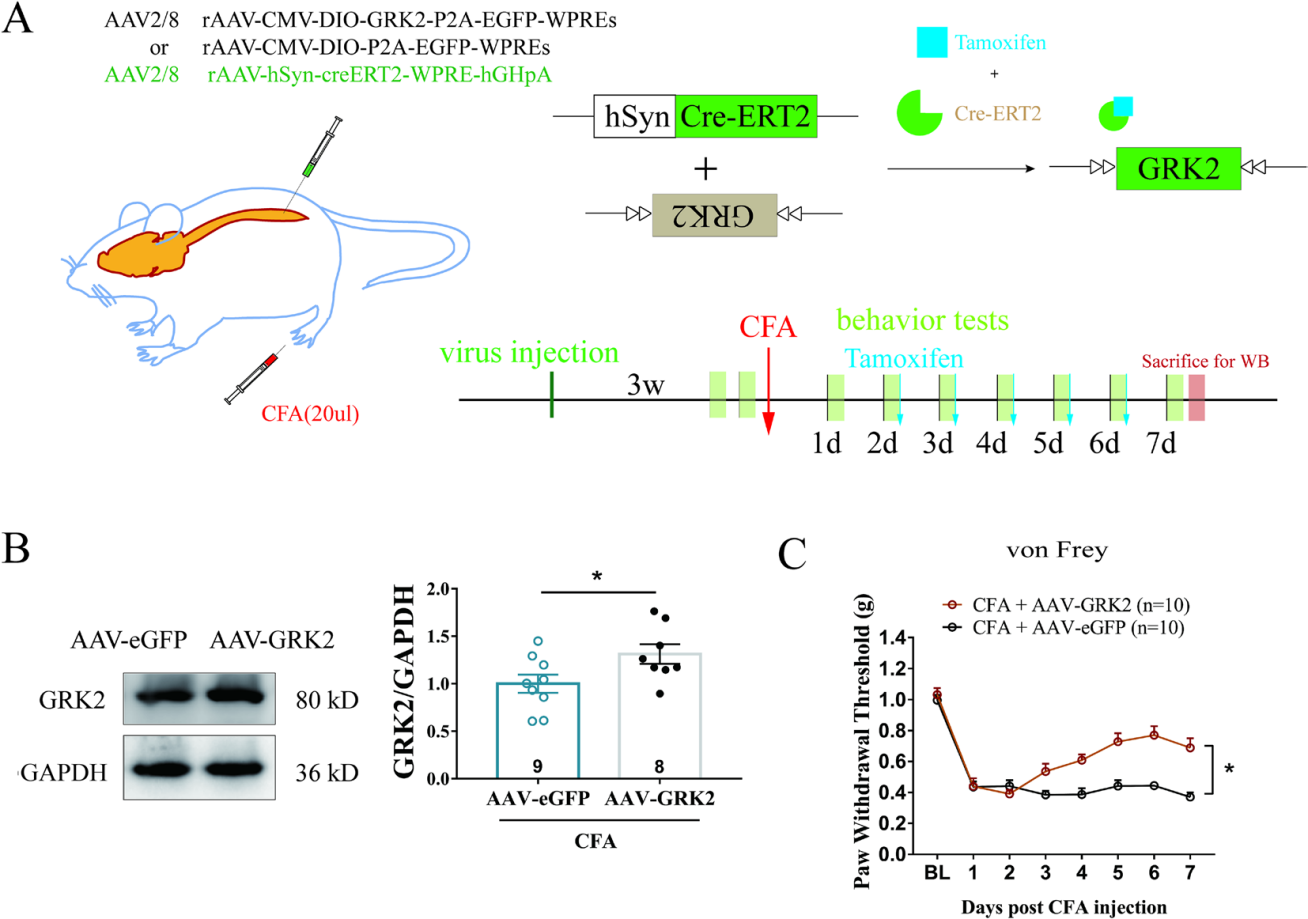
Overexpression of neuronal GRK2 in spinal cord alleviated CFA-induced pain. **A** Experimental strategy for neuronal GRK2 overexpression. Neuron specific promoter hSyn targeted Cre-ERT2 recombination activated by Tamoxifen. **B** Tamoxifen-activated Cre-ERT2 upregulated the expression of GRK2 in the spinal cord. Results are normalized to GAPDH. n = 8 for AAV-eGFP group and AAV-GRK2 group. Data are expressed as means ± SEM. *p < 0.05. Student’s *t* test was used for comparisons. **C** The mechanical threshold to von Frey stimuli in the AAV-GRK2 mice was significantly increased as compared with that of the AAV-eGFP mice. n = 10 for AAV-eGFP group and AAV-GRK2 group. Data are expressed as means ± SEM. The statistical method is repeated measures ANOVA. *p < 0.05

To further clarify whether neuronal GRK2 is involved in analgesia, neuronal GRK2 in the spinal cord was upregulated by intraspinal injection of AAV vector with neuron specific promoter hSyn and CRE enzyme induced by tamoxifen (AAV2/8 carrying hSyn-CreERT2), (*p < 0.05, Fig. 4A, B), rAAV-CMV-DIO-GRK2-P2A-EGFP-WPREs (AAV-GRK2 for short) and control group rAAV-CMV-DIO-P2A-EGFP-WPREs (AAV-eGFP for short) (n = 8 for AAV-eGFP group and AAV-GRK2 group). The results showed that after the CFA injection and Tamoxifen induction, the mechanical paw withdrawal threshold in AAV-GRK2 mice was significantly increased compared with that of the AAV-eGFP mice (*p < 0.05, Fig. 4C) (n = 10 for AAV-eGFP group and AAV-GRK2 group), indicating that neuronal GRK2 overexpression in the spinal cord prevented the development of CFA-induced persistent hyperalgesia.

## Discussion

Chronic inflammatory pain is one of the most common symptoms that seriously affects patients' quality of life. GRK2 has been reported as a key regulator in the development and maintenance of pain, and restoring GRK2 in nociceptors could prevent and relieve pain [13, 15]. However, knowledge about the role of GRK2 in the spinal cord in EA analgesia remains limited. In this study, we uncovered that the restoration of GRK2 in the spinal cord contributed to the analgesic effect of EA on inflammatory pain. Specifically, by using the mice model of CFA-induced inflammatory pain, GRK2 in spinal dorsal horn was significantly decreased. EA treatment significantly increased the GRK2 level in CFA treated mice, and alleviated CFA-induced mechanical allodynia and microglial activation and neuroinflammation in the spinal cord. The functional importance of spinal GRK2 in EA analgesia was attested by our findings that non-cell specific downregulation or neuronal downregulation of GRK2 in the spinal cord inhibited the effects of EA on mechanical allodynia, microglial activation and neuroinflammation in CFA treated mice. Based on our new findings, we propose that reestablishing GRK2 is an important mechanism involved in EA analgesia on inflammatory pain.

The neuroinflammation and neuroimmune activation are thought to mediate the central sensitization and the development of hyperalgesia and allodynia [19, 38]. Known as the macrophage in the central nervous system, microglia play an important role in the maintenance of the homeostasis of the CNS [39]. In the present study, a significant increase of Iba1, along with the mRNA expression of TNF-α, IL-1β and iNOS, indicating microglial activation and inflammatory response, were observed after the intraplantar CFA injection. This microglial activation and neuroinflammation in the spinal cord by CFA were significantly inhibited by EA treatment. On the other hand, the mRNA expression of IL-4, IL-10 and CD206 significantly decreased after the CFA injection, and EA up-regulated them in this study. For sham EA treatment, the acupuncture needles were inserted into the same acupoints at the same depth as the EA treatment in our study. It is well-known that the acupoints are richly innervated by peripheral nerve endings [1]. It has also been reported that manual acupuncture produces significant analgesic effects on pain [40]. Though shamEA shows only a slight effect on iNOS and there is no significant difference between EA and shamEA, our results still show that EA significantly decrease iNOS mRNA in comparison with the CFA group. All these suggested that EA was able to affect the microglial functions in the spinal cord in CFA-induced inflammatory pain.

Microglia has been implicated in the acquisition and/or the development of most neurological disorders [39], including Alzheimer’s disease [41], Parkinson’s disease [42, 43], stroke [44], and some chronic pain conditions [27, 45]. There is mounting evidence indicating that the therapeutic strategies for those neurological diseases may induce reduction of neuroinflammation [27, 46]. For example, in the mouse model of cisplatin-induced peripheral neuropathy, inhibition of the triggering receptor expressed on myeloid cells 2 (TREM2) by an anti-TREM2 neutralizing antibody induced a decrease of TNF-α, IL-1β, IL-6, iNOS, and CD16 in the spinal cord and an increase of IL-4, IL-10 and CD206 [27]. Inhibiting microglial activation and neuroinflammation has been proposed as a key therapeutic target for the development of neuroprotective treatments for neurological diseases [47]. In this regard, the data obtained from the present study indicate that the inhibition of microglial activation and neuroinflammation in the spinal dorsal horn might be a promising therapeutic target of electroacupuncture.

The importance of GRK2 in nociceptors in pain relief has already been demonstrated by earlier studies [13, 15, 16]. In carrageenan-primed mice, prostaglandin E2 (PGE2) induced prolonged hyperalgesia as compared with the WT mice. Intraplantar administration of herpes simplex virus (HSV) -GRK2 amplicons to increase GRK2 protein levels in dorsal root ganglion (DRG) neurons prevented the prolongation of PGE2-induced hyperalgesia in carrageenan-primed mice [16]. In the well-established model of CFA-induced inflammatory pain, GRK2 level was decreased in the spinal cord after intraplantar injection of CFA. Moreover, increasing the neuronal GRK2 level by intraspinal administration of AAV vector with neuronal promotor during ongoing inflammatory pain significantly inhibited CFA-induced inflammatory pain. Our results indicated that targeting GRK2 in the spinal cord might be the main manner of how EA affected inflammatory pain intensity and certain mediators after inflammation had already developed and pain was present.

It has been reported that GRK2 is expressed in neuron, astrocyte and microglia, and GRK2 in different cells has different effects on different inflammatory pain models [12, 14, 48, 49]. In mice models of hyperalgesia induced by carrageenan, CCL3 or IL-1β, reduction of GRK2 in microglia/macrophage/granulocyte is sufficient to cause a prolonged hyperalgesia with a duration similar to that observed in mice with low GRK2 in all cells, but reduction of GRK2 in sensory neuron is not sufficient to mimic the prolonged hyperalgesia observed in mice with low GRK2 in all cells [48]. Interestingly, in the CFA-treated mice, CFA induced the decrease of GRK2 expression while EA treatment markedly increased the GRK2 level in the spinal cord. Upregulation of neuronal GRK2 in the spinal cord significantly increased the mechanical threshold in CFA-treated mice. Downregulation of GRK2 or downregulation of neuronal GRK2 in the spinal cord both significantly inhibited EA analgesia on mechanical allodynia and inhibited the regulatory effect of EA on microglial activation and neuroinflammation in the spinal cord. Our results suggested that the neuronal GRK2 in the spinal cord played an important role in EA analgesia on CFA-induced inflammatory pain. Low GRK2 in neuron is sufficient to cause a prolonged hyperalgesia induced by epinephrine with a duration similar to that observed in mice with low GRK2 in all cells [48, 49]. All these indicated that the neuronal GRK2 and microglial GRK2 may have different roles in different models of inflammatory pain. The case being so, additional investigations may be needed to evaluate the role of microglial GRK2 in EA analgesia in the future. The present study suggested that GRK2, especially the neuronal GRK2 in the spinal cord, might be an important target of EA for its analgesic effect on inflammatory pain.

**Fig. 5:**
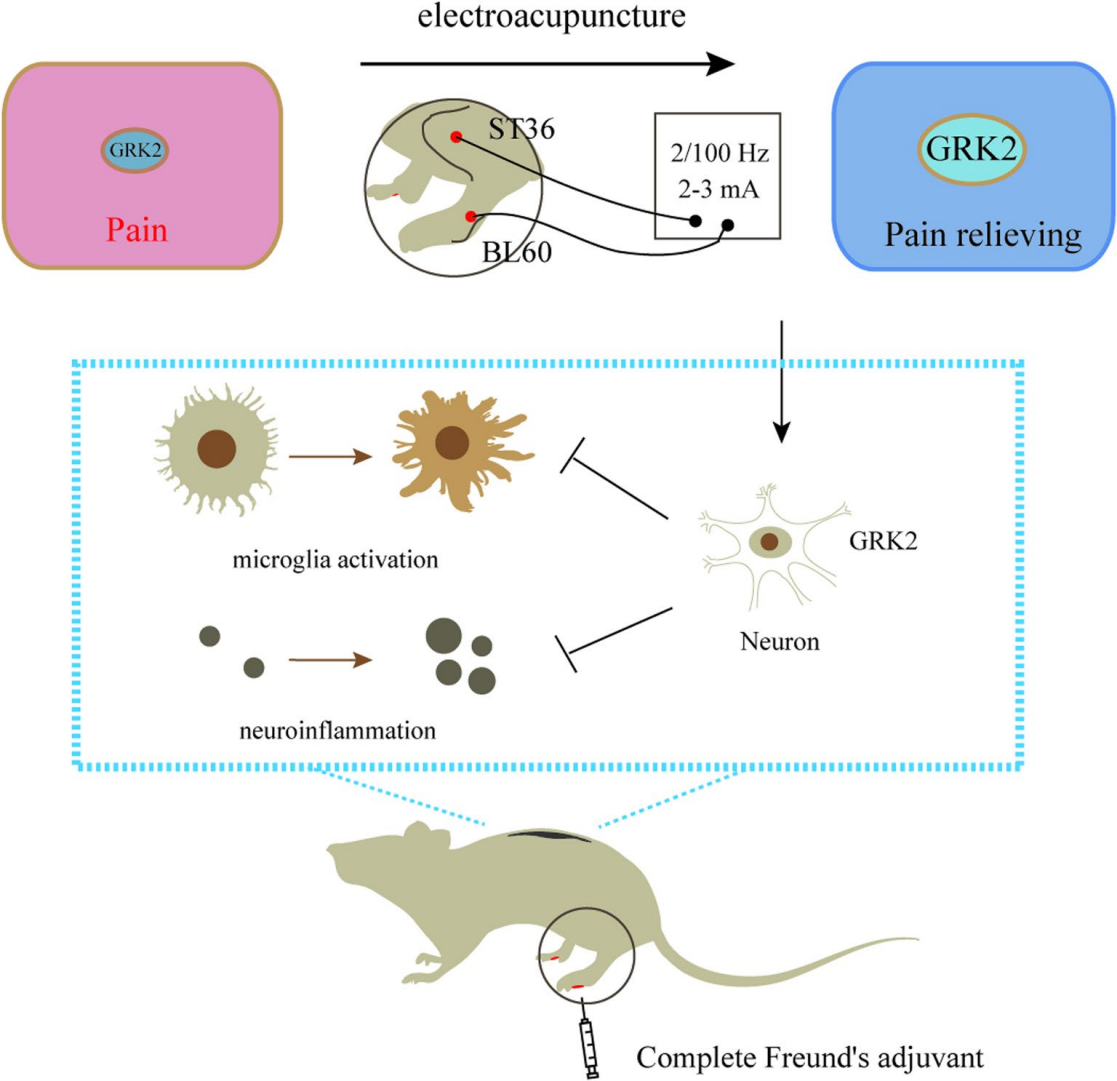
Schematic representation of the working model. CFA decreased the expression of GRK2 and induced microglial activation and neuroinflammation in the spinal cord and finally induced the development of pain. In contrast, EA regained the GRK2 probably by increasing neuronal GRK2, and then suppressing microglial activation and reducing neuroinflammation which promoted pain relief

As a result of the integration of EA signal and nociceptive signals in the spinal cord, our data demonstrated that in the mice model of inflammatory pain, restoration of the GRK2 in the spinal cord, suppressing microglial activation and reducing of neuroinflammation, are important mechanisms for EA analgesia, and the analgesic effect of EA is achieved, at least in part, through the spinal neuronal GRK2 (Fig. 5). Consistent to present work, our recent study also demonstrated the contribution of the neuronal GRK2 in the preventive effect of EA on the development of cisplatin-induced peripheral neuropathy [50]. Here, we provided a new experimental basis for the EA treatment of inflammatory pain.

## Conclusions

This research suggested that EA treatment restored GRK2 probably by increasing neuronal GRK2, and then suppressing microglial activation and reducing neuroinflammation in the spinal cord which promoted inflammatory pain relief. Our findings provided a new experimental basis for the EA treatment of inflammatory pain.

## Materials and methods

### Experimental animals

The experiments were performed on adult male C57Bl/6 mice at the age of 8–10 weeks. Animals were obtained from Shanghai SLAC Laboratory Animal Co., Ltd. China. They were housed under a 12 h light/dark cycle at a room temperature of 23 ± 0.5 °C with free access to food and water. Prior to experimental manipulation, mice were habituated in the animal room for at least one week after delivery. The experiment was designed as a single study, approved by local ethical committee at School of Basic Medical Sciences, Fudan University, People’s Republic of China (Agreement No. 20140226-087).

### Induction of CFA-induced inflammatory pain

According to the previous study [51], to establish the CFA-induced inflammatory pain model, 20 μl CFA (Sigma, F5881-10ML) was subcutaneously injected into the plantar surface of hind paw to induce an inflammatory response in mice. Normal saline with the same volume was used in the control group.

### Behavioral tests: up-and-down method

All experiments were conducted in accordance with the National Institutes of Health Guide for the Care and Use of Laboratory Animals and the Ethical Issues of the International Association for the Study of Pain. For all behavioral testing, the mice were exposed to the testing environment without any stimulation for 1–2 h per day for 2 days before the formal testing and the investigators were blinded to the treatment.

Mechanical allodynia was measured using a series of von Frey hairs (0.02, 0.04, 0.07, 0.16, 0.4, 0.6, 1.0 and 1.4 g) (Stoelting, Wood Dale, Illinois, USA) as described previously [27]. Briefly, each mouse was placed individually into a plexiglass chamber for 30-min acclimation. Then a von Frey hair was applied and held for approximately 3–4 s with a 10-min interval between applications. A trial began with the application of 0.16 g von Frey hair. The hair force was increased or decreased according to the response. A positive response was defined by a brisk withdrawal of the hind paw upon stimulation. The test contained five more stimuli after the first change in response occurred. Final score was converted to a 50% von Frey threshold using the Dixon up-and-down paradigm [52].

### EA treatment

As described previously [53], during EA treatment, the mice were placed in a specially designed holder, and the trunk of mice was kept motionless while the head and four limbs were left free to move. Two pairs of stainless-steel needles (0.16 mm in diameter) were inserted into ‘Kunlun’ (BL60, at the ankle joint level and between the tip of the external malleolus and tendo calcaneus) and ‘Zusanli’ (ST36, 5 mm lateral to the anterior tubercle of tibia) at a depth of 2 mm and 3 mm, respectively. Alternating trains of dense-sparse frequencies (100 Hz and 2 Hz each for 3 alternately), 1 mA and 2 mA for 15 min, respectively, were delivered by Han’s Acupoint Nerve Stimulator (LH202, Huawei Co. Ltd., Beijing, China). EA treatment, starting on the first day after the CFA injection, was applied once a day in the EA treatment group. Mice in sham-EA group were restrained the same way and the needles were inserted into ST36 and BL60 at a depth of 3 mm and 2 mm in the muscle without electric stimulation.

### Intraspinal AAV virus injections

The virus injection was applied 3 weeks before the CFA injection. Mice were anesthetized and then placed in a motorized stereotaxic frame, and the vertebral column was immobilized using a pair of spinal adaptors to expose the L4-L5 lumbar segment. The glass capillary for Nanoliter 2010 (outer diameter of 1.14 mm, inner diameter of 0.53 mm) was used to be inserted into the dorsal spinal cord at a depth of 200–300 μm. The rate of injection (60 nl/min) was controlled using a motorized perfusion system (Nanoliter 2010, World Precision Instruments). The glass capillary was left in place for 5 min after the injection. Wounds were sutured, and the animals were allowed to recover on a heat mat. AAV virus vectors used in this study are provided by OBiO Technology (Shanghai, China) and BrainVTA (Wuhan, China). Mice received 5 daily 100 μl injections of tamoxifen (1 mg, i.p.) (Sigma, T5648-1G) to induce CRE activity; tamoxifen was first dissolved in 95% ethanol and then diluted to its final concentration in corn oil [54]. For GRK2 overexpression experiment, two viruses were used, one (AAV2/8 carrying hSyn-CreERT2) was the virus expressing CRE enzyme induced by tamoxifen and the other (AAV2/8 carrying CMV-DIO-GRK2-2A-EGFP) was the virus expressing GRK2 dependent on CRE enzyme. For overexpression of the neuronal GRK2 in the spinal cord, mice were injected with a 2:1 volume mixture of an AAV2/8 carrying CMV-DIO-GRK2-2A-EGFP and an AAV2/8 carrying hSyn-CreERT2 (OBiO Technology Co. Ltd., Shanghai, China). Tamoxifen was first dissolved in 95% ethanol and then diluted to its final concentration in corn oil. For downregulation of the neuronal GRK2 expression in spinal cord, mice were injected with 600 nl of an AAV2/9 carrying GRK2 shRNA with a neuronal specific promoter Syn (OBiO Technology Co. Ltd., Shanghai, China).

### Western blotting

Briefly, the spinal dorsal horn of the L4-L6 segments was lysed in RIPA Lysis Buffer (100 μl/g, Beyotime Biotechology, P0013B), supplemented with protease inhibitors (Beyotime Biotechology, ST506). The lysate was centrifuged at 12,000 rpm for 20 min at 4 °C and the supernatant was obtained. Samples were separated with 10% acrylamide gels and then transferred onto polyvinylidene fluoride membranes by using SDS-PAGE. After blocking with 5% skim milk in tris-buffered-saline with tween (TBST) (20 mm Tris–HCl, pH 7.5, 150 mm NaCl, and 0.05% Tween-20), the membranes were incubated with rabbit anti-GRK2 (1:300, Sc-562, Santa Cruz), rabbit anti-GAPDH (1:10,000, HRP-60004, ProteinTech) or anti-β-actin (1:10,000, HRP-60008, ProteinTech) overnight at 4 °C in a shaking incubator, then washed in TBST before incubated with appropriate HRP-conjugated secondary antibody (1:10,000) for 2 h at room temperature. The objective band were detected using an ImageQuant LAS4000 mini image analyzer (GE Healthcare, Buckinghamshire, UK) and analyzed using ImageJ software (version 1.47).

### Immunofluorescence staining

Mice were deeply anesthetized with 1% pentobarbital sodium and were transcranially perfused with normal saline followed by 4% formaldehyde. The spinal cord of the L4-L6 segments were immersed in 4% formaldehyde for 4 h, 20% and 30% sucrose in 0.1 M PBS overnight at 4 °C respectively. The 30 μm-thick sections of spinal cord were prepared, then blocked in Superblock Buffer (Thermo, 37580) for 1 h at room temperature, reacted with mouse anti-NeuN (1:500, MAB377, Millipore) overnight at 4 °C, and then reacted with Alexa-594-conjugated donkey anti-mouse IgG secondary antibodies (1:1000, R37115, Invitrogen) for 2 h at room temperature. Finally, the sections were patched to the slides with sealing liquid containing DAPI (SouthernBiotech, 0100-20) and were visualized under a confocal microscope (FV10i, Olympus).

### Total RNA real-time PCR

Total RNA was isolated from L4-L6 spinal dorsal horn with RNSiso Plus (Takara, 9109) as per the manufacturer’s instructions. The samples were then analyzed with Nanodrop (Thermo). Reverse transcription was performed by using PrimeScriptTM RT reagent Kit with gDNA Eraser (Takara, RR047A) and SYBR Premix Ex TaqTM II (Takara, RR820A) for quantitative real-time qPCR analysis in the end. The housekeeping gene, GAPDH, was used as an internal reference for standardization of the analysis. The sequences of primers for each target mRNA are as follows: CD16, forward: 5′-AGA CCC AGC AAC TAC ATC C-3′, reverse: 5′-GAC TTC CTC CAG TAA TCC CT-3′; CD206, forward: 5′-GCT TCC GTC ACC CTG TAT G-3′, reverse: 5′-CTC CAC AAT CCC GAA CCT-3′; IL-4, forward: 5′-CCA TGA ATG AGT CCA AGT CC-3′, reverse: 5′-TGA TGC TCT TTA GGC TTT CC-3′; IL-10, forward: 5′-GGG AAG AGA AAC CAG GGA GA-3′, reverse: 5′-GGG GAT GAC AGT AGG GGA AC-3′; iNOS, forward: 5′-TTG ACG CTC GGA ACT GTA G-3′, reverse: 5′-GAC CTG ATG TTG CCA TTG T-3′; IL-1β, forward: 5′-GTA CAA GGA GAA CCA AGC AA-3′, reverse: 5′-CCG TCT TTC ATT ACA CAG GA-3′; IL-6 forward: 5′-CCA ATG CTC TCC TAA CAG AT-3′, reverse: 5′-TGT CCA CAA ACT GAT ATG CT-3′; TNF-α, forward: 5′-ACT CTG ACC CCT TTA CTC TG-3′, reverse: 5′-GAG CCA TAA TCC CCT TTC TA-3′; TGF-β, forward: 5′-GAC CGC AAC AAC GCC ATC TAT-3′, reverse: 5′-CAC TGC TTC CCG AAT GTC TGA-3′; GAPDH, forward: 5′-AAA TGG TGA AGG TCG GTG TG-3′, reverse: 5′-AGG TCA ATG AAG GGG TCG TT-3′. All primers used for PCR analysis are synthesized by Sangon Biotech (Shanghai) Co., Ltd.

### Statistical analysis

All statistical analyses were carried out by using the Prism7 software packages and IBM SPSS 22.0. All data are presented as mean ± standard error of the mean (SEM). A t-test was used for direct comparisons between the two groups. For multiple comparisons within one group, one-way analysis of variance (ANOVA) was adopted. Significant differences among groups were checked by two-way ANOVA. A repeated measure ANOVA was employed to compare the differences between GRK2 overexpression group and the control group. p < 0.05 was considered statistically significant.

## Supplementary Information

**Additional file 1: Fig. S1. **Western blot analysis of GRK2 from L4~L6 spinal cord at day 1, 3, 5, 7, 11 after the CFA injection (n=4). Results are normalized to GAPDH and shown as ratios to D0. The statistical method is one-way ANOVA multipule comparisons. **Fig. S2. **Downregulation of GRK2 in the spinal cord did not alter the spontaneous locomotor activity in the open field test (A)(B), the mechanical sensitivity in the von Frey test (C).Values are represented as mean ± SEM. p > 0.05. The statistical method is twoway ANOVA multipule comparisons. **Fig. S3.** (A)(B)The injection of the virus to downregulate the neuronal GRK2 did not affect the locomotive ability (open field, rotarod test). (C) Downregulation of neuronal GRK2 in the spinal cord did not alter the mechanical (von Frey) sensitivity of the mice. Values are represented as mean ± SEM. p > 0.05. The statistical method is two-way ANOVA multipule comparisons.

## Data Availability

The datasets used or analyzed during the current study are available from the corresponding author on reasonable request.

## Abbreviations

AAV: Adeno associated virus
ANOVA: Analysis of variance
CCL3: Chemokine (C–C motif) ligand 3
CD: Cluster of differentiation
CFA: Complete Freund’s adjuvant
CNS: Central nervous system
DRG: Dorsal root ganglion
EA: Electroacupuncture
ERT2: Estrogen receptor
eGFP: Enhanced green fluorescent protein
GAPDH: Glyceraldehyde-3-phosphate dehydrogenase
GPCRs: G protein coupled receptors
GRK2: G protein coupled receptor kinase 2
HSV: Herpes simplex virus
Iba1: Ionized calcium-binding adapter molecule 1
IL: Interleukin
i.p.: Intraperitoneally
iNOS: Inducible nitric oxide synthase
mRNA: Messenger ribonucleic acid
NeuN: Neuronal nuclei
NS: Normal saline
OFT: Open field test
PCR: Polymerase chain reaction
PGE2: Prostaglandin E2
SDS-PAGE: Sodium dodecyl sulphate–polyacrylamide gel electrophoresis
shRNA: Short hairpin ribonucleic acid
Syn: Synapsin
TGF-β: Transforming growth factor -β
TNF-α: Tumor necrosis factor-α
TREM2: Triggering receptor expressed on myeloid cells 2
β-actin: ACTB actin beta
