# Supplementary material for: Neuronal GRK2 regulates microglial activation and contributes to electroacupuncture analgesia on inflammatory pain in mice
Source: Biol Res. 2022 Feb 3;55:5. doi: 10.1186/s40659-022-00374-6 (PMC8812183; doi:10.1186/s40659-022-00374-6)
Supplement: Supplementary file 1 — Additional file 1: Fig. S1. Western blot analysis of GRK2 from L4~L6 spinal cord at day 1, 3, 5, 7, 11 after the CFA injection (n=4). Results are normalized to GAPDH and shown as ratios to D0. The statistical method is one-way ANOVA multipule comparisons. Fig. S2. Downregulation of GRK2 in the spinal cord did not alter the spontaneous locomotor activity in the open field test (A)(B), the mechanical sensitivity in the von Frey test (C).Values are represented as mean ± SEM. p > 0.05. The statistical method is twoway ANOVA multipule comparisons. Fig. S3. (A)(B)The injection of the virus to downregulate the neuronal GRK2 did not affect the locomotive ability (open field, rotarod test). (C) Downregulation of neuronal GRK2 in the spinal cord did not alter the mechanical (von Frey) sensitivity of the mice. Values are represented as mean ± SEM. p > 0.05. The statistical method is two-way ANOVA multipule comparisons. [file 40659_2022_374_MOESM1_ESM.pdf]

Additional file 1: Fig. S1

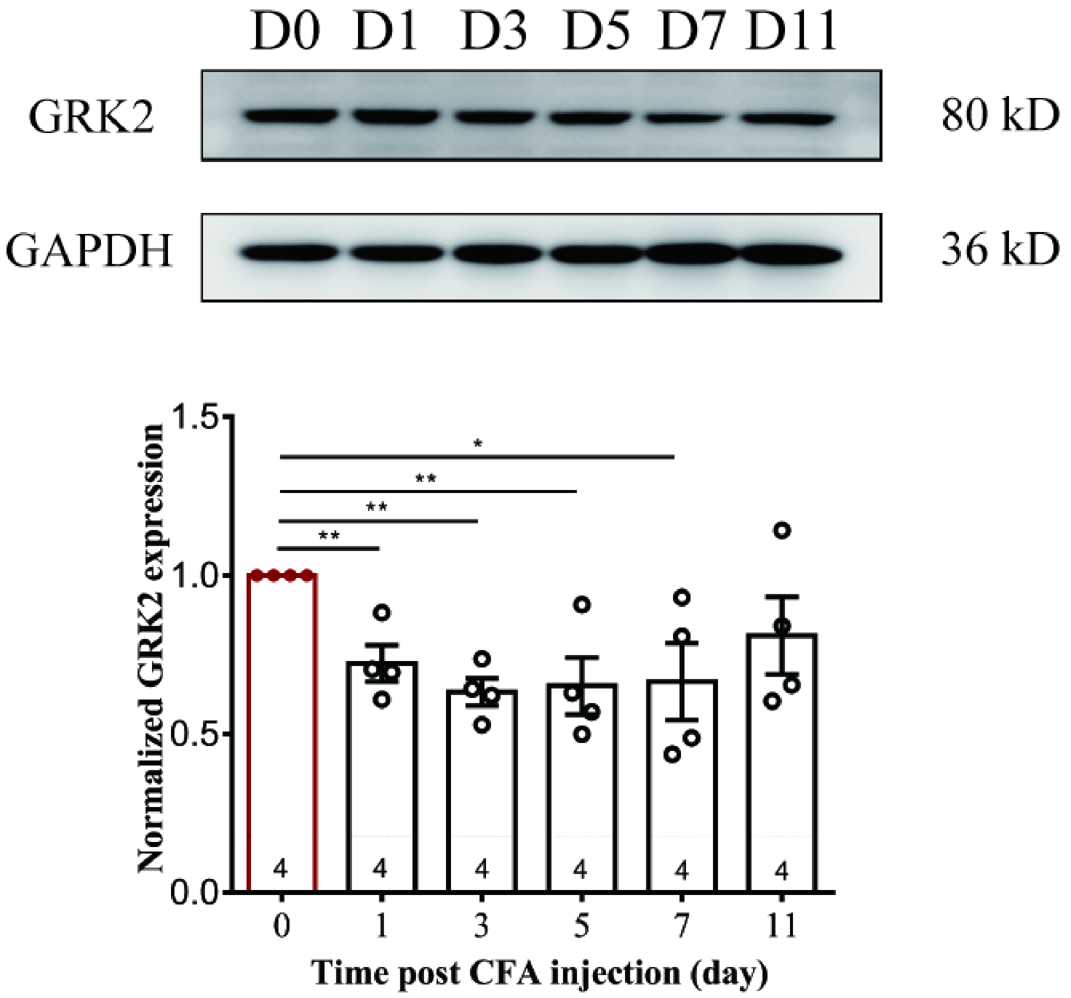

Fig. S1. The expression of GRK2 decreased in the spinal cord from the 1st to the 7th day after the CFA injection. Western blot analysis of GRK2 from L4~L6 spinal cord at day 1, 3, 5, 7, 11 after the CFA injection (n=4). Results are normalized to GAPDH and shown as ratios to D0. The statistical method is one-way ANOVA multipule comparisons.

Additional file 1: Fig. S2

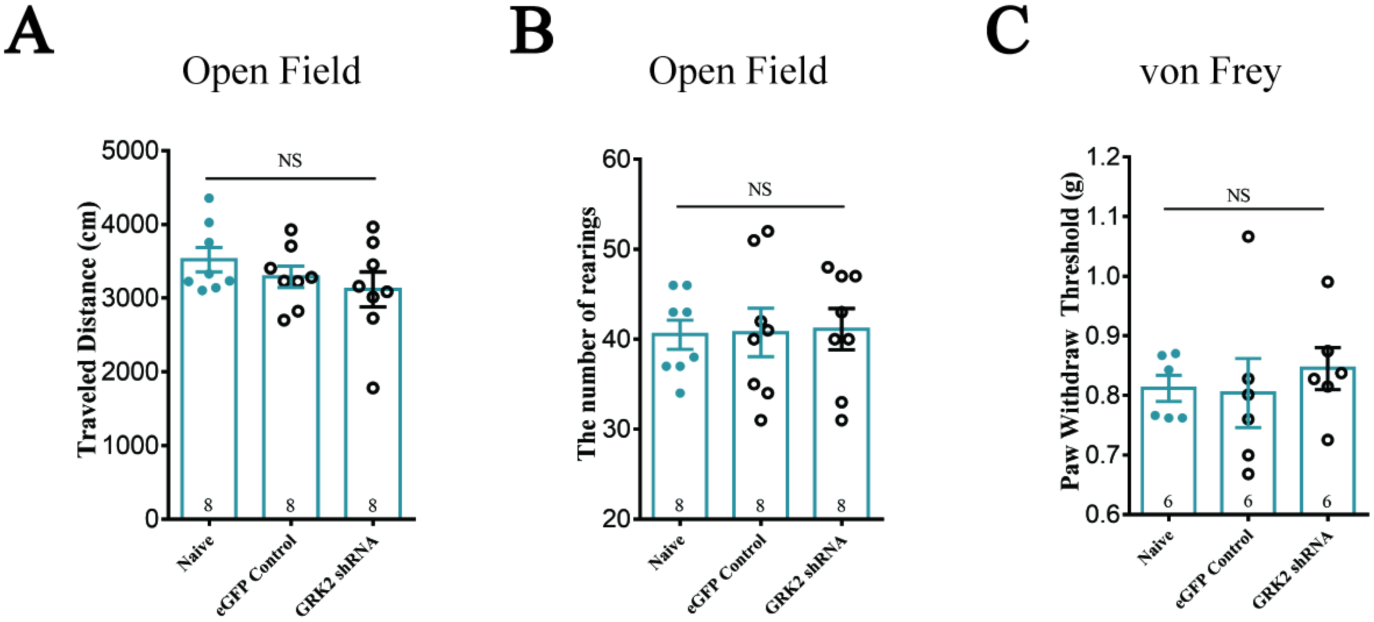

Fig. S2. The downregulation of GRK2 in the spinal cord by AAV injection did not alter locomotor activity and sensitivity of mechanical stimulation in naive mice. (**A**)(**B**) Downregulation of GRK2 in the spinal cord did not alter the spontaneous locomotor activity in the open field test. (**C**) Downregulation of GRK2 in the spinal cord did not alter the mechanical sensitivity in the von Frey test.Values are represented as mean ± SEM. $p > 0.05$. The statistical method is two-way ANOVA multipule comparisons.

Additional file 1: Fig. S3

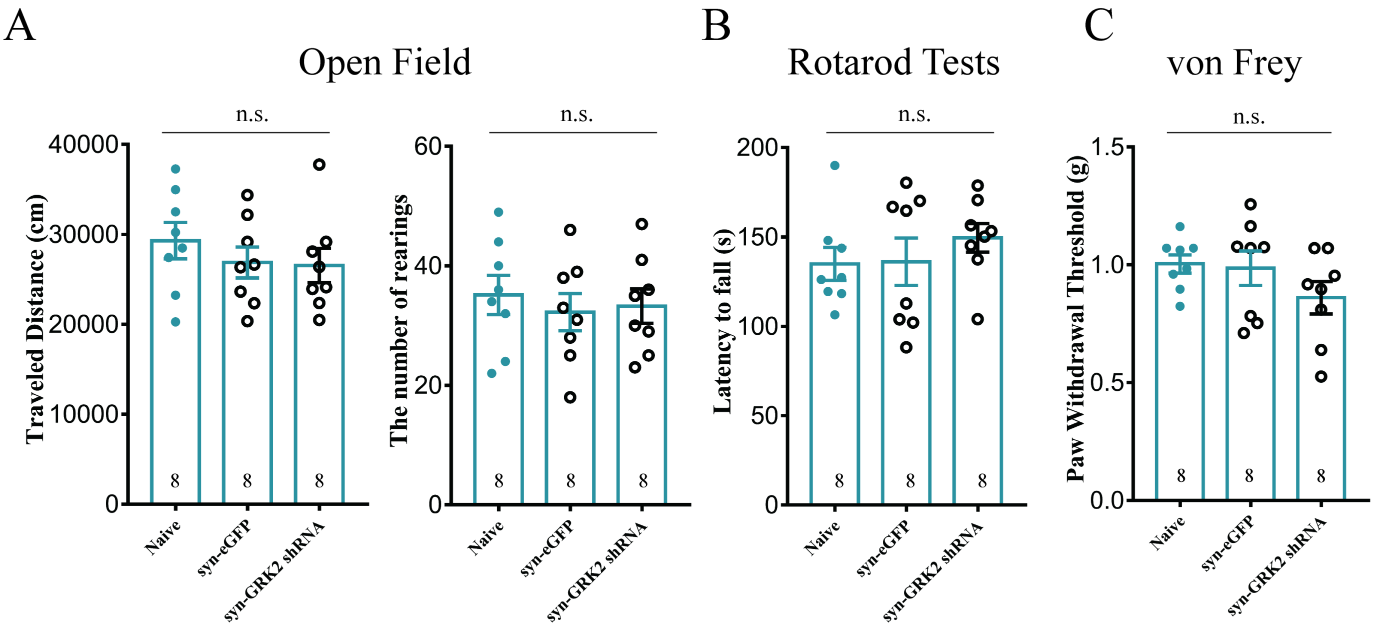

Fig. S3. Downregulation of GRK2 level in spinal neuron did not affect the locomotor activity and the sensitivity of mechanical stimulation in naive mice. (**A**)(**B**)The injection of the virus to downregulate the neuronal GRK2 did not affect the locomotive ability (open field, rotarod test). (**C**) Downregulation of neuronal GRK2 in the spinal cord did not alter the mechanical (von Frey) sensitivity of the mice. Values are represented as mean ± SEM. $p > 0.05$. The statistical method is two-way ANOVA multipule comparisons.
